# Consciousness in the Brain: An Integrative Review of Contemporary Theories

**DOI:** 10.3390/brainsci16070745

**Published:** 2026-07-14

**Authors:** Arosh S. Perera Molligoda Arachchige, Afanasy Svet, Maria Svet

**Affiliations:** 1 University of Milan, Via Festa del Perdono 7, 20122 Milan, Italy; 2 University of Geneva, 24 Rue du Général-Dufour, 1211 Geneva, Switzerland; 3 Independent Researcher, 1231 Geneva, Switzerland

**Keywords:** Global Neuronal Workspace Theory (GNWT), Integrated Information Theory (IIT), Recurrent Processing Theory (RPT), Dendritic Integration Theory (DIT), Predictive Processing (PP), Memory Theory of Consciousness (MToC)

## Abstract

Understanding consciousness remains one of the most significant challenges in neuroscience, philosophy, and cognitive science. Despite substantial advances in neuroimaging, electrophysiology, and computational modeling, a comprehensive account of the neural basis of conscious experience has yet to emerge. This review examines several leading contemporary theories of consciousness, including the Global Neuronal Workspace Theory (GNWT), Integrated Information Theory (IIT), Recurrent Processing Theory (RPT), Dendritic Integration Theory (DIT), Predictive Processing (PP), and the Memory Theory of Consciousness (MToC). The philosophical foundations, core theoretical claims, and empirical evidence supporting each framework are critically evaluated. Particular attention is given to how these theories address phenomenal consciousness—the subjective quality of experience—and access consciousness, i.e., the availability of information for cognitive control, report, and behavior. By comparing convergent and divergent predictions across theoretical perspectives, this review highlights key areas of agreement, ongoing debates, and unresolved questions in the field. The analysis suggests that consciousness is likely to involve multiple interacting neural mechanisms operating across different spatial and temporal scales, underscoring the need for continued interdisciplinary research to advance a more comprehensive understanding of conscious experience.

## 1. Introduction

Consciousness remains one of the most complex and contested phenomena in neuroscience, philosophy, and cognitive science. Its study is complicated by persistent terminological ambiguity: terms such as consciousness, awareness, phenomenal consciousness, access consciousness, subjective experience, and self-consciousness are sometimes used interchangeably and sometimes assigned distinct meanings depending on the theoretical framework. In this review, consciousness is used as the broadest term referring to the presence of subjective experience, whereas phenomenal consciousness refers specifically to the qualitative, first-person character of experience—the “what-it-is-like” aspect of seeing a color, hearing music, or feeling pain. Access consciousness refers to information that is available for reasoning, decision-making, voluntary action, and verbal report. Awareness is used more cautiously as a context-dependent term that may denote conscious perception of a stimulus but does not necessarily imply reflective awareness of oneself as the experiencing subject. Self-consciousness and self-reflection are treated as higher-order capacities involving representation of oneself as the subject of experience and are therefore not assumed to be necessary components of every conscious state [1,2,3]. Because no universal consensus exists regarding these definitions or their relationships, terminology is specified explicitly throughout this review.

Despite decades of research and numerous theories, consciousness remains poorly understood. Theoretical approaches vary widely—some emphasize the brain’s structural organization, others its dynamic activity, the integration of information, or recurrent neural processing. This diversity has resulted in a fragmented landscape of ideas [4]. However, recent integrative reviews and collaborative efforts suggest that these different theories might not be mutually exclusive but instead represent complementary levels of description or distinct facets of conscious experience.

Theories of consciousness are often categorized along multiple dimensions, including distinctions between access and phenomenal consciousness and between cognitive, biological, and physical levels of explanation. For clarity, we group perspectives here into three broad heuristic categories reflecting differing ontological assumptions, while acknowledging that this classification is not standard and that many theories span multiple categories:

(A) The dominant scientific materialist perspective holds that consciousness is not an independent entity but an emergent property arising from complex biological computations within brains and nervous systems through evolution [5,6,7]. Opinions vary regarding when consciousness first appeared—whether exclusively in humans or earlier in simpler organisms. Often seen as epiphenomenal (a secondary byproduct without causal power) or even illusory (constructing rather than directly perceiving reality), consciousness is nevertheless considered evolutionarily advantageous. In this view, consciousness is not intrinsic to the universe itself [7,8,9].

(B) In contrast, dualistic and spiritual viewpoints regard consciousness as a distinct, non-physical quality that has always existed in the universe. Philosophical dualism, religious doctrines, and spiritual traditions conceive consciousness as fundamental—sometimes as the “ground of being,” a creator, or an omnipresent divine presence. Here, consciousness may influence physical matter and behavior but lies outside the domain of scientific explanation. Related ideas include panpsychism, which attributes a form of consciousness to all matter without a clear scientific basis, and idealism, which asserts that consciousness is the only true reality, with the material world being illusory [10,11,12].

(C) A third perspective proposes that consciousness emerges from discrete physical events inherent to the universe’s fabric—proto-conscious moments governed by physical laws not yet fully understood. Biological evolution developed mechanisms to orchestrate and couple these events to neuronal activity, producing meaningful conscious experiences capable of influencing behavior. This view is exemplified by the Penrose–Hameroff “orchestrated objective reduction” (Orch OR) theory, which identifies conscious moments with quantum state reductions occurring in brain microtubules. Such quantum events are proposed to have experiential qualities, making consciousness an intrinsic feature of the universe’s operation [13,14,15,16,17,18,19,20,21].

Accordingly, this review aims to critically compare selected influential theories of consciousness across cellular, network, computational, phenomenological, and physical levels of explanation; evaluate their empirical support and limitations; examine areas of convergence and conflict; and identify unresolved questions that may guide future theory-driven research. Rather than providing an exhaustive taxonomy, we adopt an integrative, multiscale perspective to explore whether apparently competing theories may explain complementary aspects of conscious experience.

## 2. Conceptual Framework and Scope

### 2.1. Key Terminology in Consciousness Research

The study of consciousness is complicated by the absence of universally accepted definitions. Different theories may use identical terms to refer to different phenomena or, conversely, different terms for closely related constructs. For clarity, Table 1 presents the operational meanings adopted throughout this review. These definitions are intended as a pragmatic conceptual framework rather than claims of universal consensus and should be interpreted in the context of the theoretical distinctions discussed in subsequent sections.

### 2.2. Scope and Organizational Framework of This Review

While several recent reviews have systematically categorized theories of consciousness, the present review adopts a complementary approach [22,23,24,25]. Rather than aiming to provide an exhaustive taxonomy, we focus on a selected set of influential and conceptually distinct frameworks spanning multiple explanatory levels, including cellular mechanisms, large-scale neural networks, computational principles, memory-related accounts, phenomenological frameworks, and physical or ontological proposals.

The included theories were selected according to three broad criteria: (i) engagement with neuroscientific, computational, clinical, or philosophical debates concerning consciousness; (ii) conceptual distinctiveness in explaining phenomenal experience, conscious access, or their underlying mechanisms; and (iii) relevance to contemporary discussions regarding the neural correlates, organization, and possible physical basis of consciousness.

The frameworks examined differ substantially in empirical grounding, methodological development, and scientific status. Established neuroscientific theories such as Global Neuronal Workspace Theory, Recurrent Processing Theory, Predictive Processing, and Integrated Information Theory have generated testable predictions and have been extensively investigated using neuroimaging, electrophysiology, computational modeling, and clinical studies. Other frameworks, including Dendritic Integration Theory and quantum- or field-based proposals, remain more preliminary or hypothesis-generating. Philosophical approaches such as dualism, panpsychism, and idealism address important ontological questions concerning subjective experience and the mind–body relationship but generally do not provide experimentally validated neural mechanisms.

The purpose of including these diverse perspectives is therefore not to imply equivalent evidential support but to illustrate the breadth of contemporary attempts to explain consciousness. Throughout this review, interpretations are considered in relation to the available empirical evidence and the methodological status of each framework. The proposed classification should be understood as a pragmatic organizational structure rather than an exhaustive taxonomy, particularly because several theories span multiple categories or resist simple classification (Table 2).

The proposed classification should therefore be understood as a pragmatic organizational framework rather than an exhaustive taxonomy of contemporary consciousness theories. Several additional proposals—including electromagnetic-field, endogenous-field, and other large-scale physical-field accounts—fall partly outside these categories and remain active, although generally speculative, areas of theoretical investigation.

## 3. Theoretical Frameworks of Consciousness

### 3.1. Neurocomputational and Functional Theories of Consciousness

One of the most influential models of consciousness is the Global Neuronal Workspace Theory (GNWT), developed primarily by Stanislas Dehaene and colleagues [26]. This theory postulates that conscious access occurs when information is broadcast to a global workspace composed of long-range connections linking the prefrontal, parietal, and cingulate cortices. According to GNWT, various specialized processors operate unconsciously in parallel. When a piece of information becomes the focus of attention and is amplified sufficiently, it enters the global workspace, becoming available for verbal report, decision-making, and intentional action [26]. This “ignition” process is thought to be marked by late, sustained neural activity, particularly in the P3b component observed in event-related potentials. Empirical support for GNWT comes from studies using masked priming, binocular rivalry, and attentional blink paradigms [27,28,29]. Neuroimaging studies show that conscious perception is consistently associated with widespread frontoparietal activation, while unconscious processing remains confined to local sensory areas [30,31]. GNWT conceptualizes consciousness as a functional process associated with global information broadcasting, rather than denying the existence of subjective experience [26]. However, it remains debated whether frontoparietal activation reflects consciousness itself or processes related to reporting, attention, and task demands (e.g., Tsuchiya et al., 2015) [32].

The Higher-Order Theory (HOT) suggests that consciousness arises from meta-representations—representations of other mental representations—occurring at higher levels of processing. For instance, a visual scene such as a painting may first be represented in the visual cortex simply as patterns of color, like red and green areas. It becomes a conscious experience only when this lower-level information is transmitted to higher brain regions, particularly the prefrontal cortex, enabling self-reflective awareness, such as the thought, “*I am seeing a painting*” [33].

The Memory Theory of Consciousness (MToC) argues that phenomenal consciousness evolved as part of episodic and other explicit memory systems, including sensory, working, and semantic memory [34,35]. This framework accounts for phenomena such as postdictive effects, where the conscious perception of a stimulus can be influenced by events occurring after the stimulus is presented. According to MToC, the cerebral cortex and hippocampus work together to sequentially integrate and time-stamp parallel unconscious processes, producing a coherent, linear flow of conscious experience. In this view, consciousness is essentially a form of memory, preceded by unconscious stages of perception, decision-making, or action [34,35]. Furthermore, it holds that different cortical regions generate distinct aspects of consciousness, each with its own specific neural correlates. This perspective is especially relevant for understanding conditions like delirium and amnesia, where the integrity of memory systems is compromised [34,35,36]. However, this view has been critiqued for potentially conflating memory processes with conscious experience itself (Hogendoorn, 2023), highlighting the need for clearer distinctions between perception and postdictive reconstruction [36].

In contrast, the Integrated Information Theory (IIT), pioneered by Giulio Tononi, begins with phenomenology [37]. It asserts that consciousness corresponds to the capacity of a system to integrate information [38]. This capacity is quantified by a value called Phi (Φ), which represents the degree to which the whole system’s information exceeds the sum of its parts [39]. IIT posits that high Φ systems are both highly differentiated and highly integrated, leading to a rich repertoire of possible states [40]. According to IIT, the physical substrate of consciousness resides in the “posterior hot zone” of the cortex, including the temporo-parieto-occipital junction, which shows high integration and complexity [41].

Interpretations of lesion studies remain complex and theory-dependent. While some frameworks emphasize distributed cortical contributions, others highlight specific regional or network-level roles. Consequently, lesion evidence does not straightforwardly adjudicate between competing theories but instead constrains their explanatory scope [37,38,39,40,41]. The IIT has inspired a range of empirical studies attempting to measure Φ through techniques such as the Perturbational Complexity Index (PCI), which involves stimulating the cortex with transcranial magnetic stimulation (TMS) and recording the complexity of the EEG response. IIT has been influential in discussions about disorders of consciousness, such as vegetative and minimally conscious states, where PCI can serve as a biomarker [37,38,39,40,41].

Recent large-scale adversarial collaborations have provided important empirical constraints on leading theories of consciousness. Notably, the COGITATE consortium (2025) conducted a preregistered multimodal study (fMRI, MEG, and intracranial EEG; n = 256) directly comparing predictions from GNWT and IIT. The findings challenged key aspects of both frameworks: sustained posterior connectivity patterns predicted by IIT were not consistently observed, and GNWT predictions of late global “ignition” at stimulus offset and strong prefrontal representation of conscious content were only partially supported. These results highlight the importance of large-scale, theory-driven experimental tests and suggest that no single framework currently provides a complete account of consciousness. Ongoing efforts, including published responses and parallel collaborations such as INTREPID, continue to refine these debates [42,43,44].

Local recurrent theories broadly propose that recurrent processing within sensory cortices may support conscious perception without requiring global prefrontal broadcasting, i.e., conscious experience can emerge from recurrent processing within sensory cortices alone. In this model, parietal and frontal brain regions are involved not in generating consciousness itself but rather in enabling verbal reports and judgments about the stimulus [45,46]. Recurrent Processing Theory (RPT), most prominently associated with Victor Lamme, represents an influential formulation of this family of theories [47]. It argues that recurrent (feedback) interactions within sensory cortices are sufficient for consciousness, even in the absence of prefrontal involvement [48]. According to RPT, early feedforward sweeps through the sensory hierarchy are not conscious per se. Consciousness arises when there is sustained, recurrent processing between higher and lower areas, allowing for contextual modulation and perceptual binding [49]. This theory is supported by visual masking experiments and single-cell recordings in primates showing that recurrent activity correlates more strongly with perceptual awareness than feedforward responses. Unlike GNWT, RPT emphasizes phenomenal consciousness and downplays the role of report and higher cognitive functions [47,48,49].

The Dendritic Integration Theory (DIT), more recently proposed by Matthew Larkum and others, addresses consciousness at the cellular level. It focuses on the role of layer 5 pyramidal neurons, which possess dendrites that receive inputs from both lower and higher cortical areas [50]. According to DIT, these neurons act as coincidence detectors, integrating inputs from the apical and basal dendrites to produce bursts of activity that may underpin conscious experience. The theory suggests that conscious perception depends on the simultaneous activation of cortico-cortical and thalamo-cortical loops, with dendritic processing playing a central role. DIT represents a promising attempt to link cellular mechanisms to conscious phenomena, though it remains largely theoretical and in need of empirical validation [51].

Predictive Processing (PP) and Neurorepresentationalism (NREP) offer yet another lens. These theories conceptualize the brain as a hierarchical prediction machine that continuously generates and updates models of the world [52,53,54]. Consciousness, in this framework, may emerge from the precision-weighting of prediction errors and the updating of generative models. PP accounts for a wide array of phenomena, including illusions, hallucinations, and the effects of attention [54]. While not originally intended as a theory of consciousness per se, it has been extended to explain both the contents and levels of consciousness by linking them to model complexity and error minimization [55]. Some proponents argue that phenomenal consciousness corresponds to the content of high-level predictions, while access consciousness relates to the modulation of lower-level sensory prediction errors [52,53,54,55,56].

Category theory offers a formal insight called the Yoneda Lemma, an alternative, relational approach that formalizes entities in terms of their relationships to all others within a given domain [57]. According to the Yoneda Lemma, an object is fully determined by the pattern of relations it holds with every other object in its category. Applied to consciousness, this suggests that a given conscious state can be completely characterized by the lawful transitions into and out of it from all other possible states, rather than by its internal constitution alone. By defining categories of consciousness—whether in terms of levels or contents—we can exhaustively describe consciousness through its relational structure. This framework offers a potential gold standard for formalizing empirical research, such as mapping the structure of color qualia at the fovea and periphery, and for rigorously testing competing theories of consciousness by comparing their predicted relational patterns against observed data [57].

### 3.2. Philosophical and Non-Physicalist Perspectives on Consciousness

Under this category, one of the most important theories is Eccles’s dualist interactionism, a theory in the philosophy of mind that posits the existence of two distinct substances—the mental (mind or soul) and the physical (body/brain)—which interact causally [58,59]. Developed by neurophysiologist Sir John Eccles to account for consciousness and free will, the model proposes that mental events can influence physical processes and vice versa. Eccles introduced the concepts of *psychons*—units of conscious experience—and *dendrons*—neural assemblies in the cortex with which psychons interact. He suggested that psychons could modulate the probability of neuronal firing via quantum effects at synapses, providing a potential mechanism for mind–brain interaction without overtly violating physical laws [60]. This framework supports a libertarian view of free will, where the mind can exert top-down control over voluntary actions. Eccles argued that dualist interactionism offers a more complete causal explanation of mental phenomena than monistic materialism, which he saw as struggling to bridge the explanatory gap. However, the theory has been criticized for its speculative nature, the vague definition of key constructs, difficulties in empirical testing, and potential conflicts with conservation laws. Despite these challenges, it remains a landmark example of interactionist dualism in the modern philosophy of mind [60]. These proposed mechanisms have been widely critiqued on physical and theoretical grounds, including concerns about the plausibility of quantum effects at synapses and the lack of empirical support [61,62].

Contemporary discussions of non-physicalist approaches also include more analytically grounded frameworks such as property dualism and panpsychism, which attempt to reconcile subjective experience with physicalist science without invoking strong interactionist claims.

### 3.3. Science with Incomplete Laws: Consciousness as an Essential but Not Yet Fully Understood Component of Physical Reality

The Orch-OR theory proposes that consciousness arises from quantum processes within microtubules—tiny cylindrical structures inside neurons. These microtubules are thought to sustain quantum superpositions involving the states of tubulin proteins, representing multiple potential informational arrangements simultaneously [63,64]. Conscious moments occur when these quantum superpositions undergo Objective Reduction (OR), a physical collapse caused by an instability in spacetime geometry related to gravity, as proposed by Penrose [20]. Each such collapse corresponds to a discrete event of awareness, and the continuous flow of consciousness is seen as a sequence of these collapses, much like frames in a film. The brain “orchestrates” these quantum states by synaptic inputs and memory patterns, biasing the possible outcomes so that the collapse produces meaningful and experience-based results rather than random noise. The outcome of the collapse influences whether the neuron fires, effectively embedding the quantum event into classical neural processing, which links subjective awareness with physical brain activity. This theory contrasts with classical neuroscience, which views consciousness as emerging purely from complex neural computations. Instead, Orch-OR argues that consciousness fundamentally depends on a non-computable physical process rooted in quantum gravity and spacetime structure [65,66,67]. The theory also explains the timing of conscious moments, suggesting they occur roughly every 25–100 milliseconds, matching observed brain wave rhythms, a timescale its proponents argue is consistent with gamma-band activity, although this relationship remains debated. Although the warm, wet brain was traditionally thought too noisy for quantum coherence, biological evidence from processes like photosynthesis and bird navigation shows that quantum coherence can exist at body temperature. Microtubules may provide a shielded environment supporting this coherence, and experiments have observed resonant vibrations and high-frequency oscillations in microtubules that could facilitate quantum processing in the brain [68,69].

Despite its conceptual appeal, Orch-OR remains controversial. Critics argue that quantum coherence may be difficult to sustain at biologically relevant timescales in the warm and noisy environment of the brain. Furthermore, many proposed quantum mechanisms have not yet been experimentally demonstrated in neural tissue, and direct evidence linking microtubule quantum processes to conscious experience remains limited. Consequently, Orch-OR should currently be regarded as a speculative but influential hypothesis rather than an established neuroscientific theory.

### 3.4. Field-Based and Other Physical Frameworks

A further group of proposals attributes consciousness partly or primarily to large-scale physical fields generated by, or interacting with, neural activity. Electromagnetic-field theories, for example, propose that the brain’s endogenous electromagnetic field may contribute to the integration or unification of distributed neural information. Other field-based or ontological frameworks invoke broader physical organizational principles that cannot be readily classified as conventional neurocomputational emergence, philosophical dualism, or quantum state-reduction theories. At present, these approaches remain heterogeneous and generally possess substantially less direct experimental support than established neuroscientific frameworks such as GNWT, RPT, or predictive processing. They are therefore best regarded as emerging or speculative proposals rather than established theories of consciousness. Their inclusion here is intended to acknowledge the breadth of contemporary theorizing rather than imply evidential equivalence among competing frameworks [70,71,72].

## 4. Empirical and Clinical Correlates

Across these theoretical frameworks, a recurring theme is the identification of neural correlates of consciousness (NCCs). These are the minimal neural mechanisms sufficient to support a specific conscious experience. EEG and ERP components like the P300, visual awareness negativity (VAN), and the Pe component have been studied extensively [73,74,75]. The VAN, for example, is an early marker occurring around 200 ms post-stimulus and is thought to index perceptual awareness [76]. The P300, on the other hand, is associated with post-perceptual processes such as working memory updating and decision-making. These components have been used to distinguish between conscious and unconscious states in both healthy individuals and patients with disorders of consciousness [77]. Functional MRI studies have shown that conscious perception involves not just sensory cortices but also widespread activation in the frontoparietal network, supporting the GNWT framework. At the same time, recurrent interactions within visual cortices, emphasized by RPT, have also been validated through intracranial recordings [78]. Advances in multimodal neuroimaging, particularly hybrid PET/MRI systems, offer promising avenues for integrating structural, functional, and metabolic data, potentially improving the characterization of neural correlates of consciousness [79,80].

Clinical applications of consciousness theories are most apparent in the assessment and management of patients with brain injuries. The Coma Recovery Scale—Revised (CRS-R) and the Glasgow Coma Scale (GCS) are commonly used clinical tools, but they rely heavily on behavioral responses [81]. Advanced techniques such as PCI and resting-state fMRI offer objective, brain-based measures of consciousness. For example, patients in a vegetative state may show covert consciousness through EEG responses to verbal commands, highlighting the dissociation between behavioral unresponsiveness and neural activity [82,83,84]. Such findings underscore the importance of theoretical frameworks that can account for both conscious content and level [82].

## 5. Toward an Integrative Approach

A major challenge in consciousness research is that different theories often explain different aspects of conscious experience while employing distinct explanatory frameworks and methodologies. GNWT provides a powerful account of access consciousness and cognitive reportability but has been criticized for potentially conflating consciousness with attention, working memory, and task-related processing. IIT offers a formal framework linking consciousness to information integration and has inspired quantitative measures such as the Perturbational Complexity Index; however, the interpretation and measurement of integrated information remain controversial. RPT emphasizes recurrent sensory processing and provides a compelling account of phenomenal awareness but faces challenges in explaining higher-order cognitive access. Predictive Processing offers a unifying computational framework capable of explaining perception, attention, and hallucinations, yet its relationship to subjective experience remains incompletely specified. Similarly, DIT provides a promising cellular-level mechanism but currently lacks the breadth of empirical validation available for more established theories.

Importantly, no contemporary theory has yet achieved broad consensus as a complete explanation of consciousness. Recent large-scale adversarial collaborations, including the COGITATE project, have demonstrated that key predictions from leading frameworks are only partially supported by experimental data. These findings suggest that consciousness may not be adequately explained by a single mechanism operating at one spatial or temporal scale. Instead, conscious experience may emerge from interactions among cellular processes, recurrent neural dynamics, large-scale network integration, predictive computations, and memory-related mechanisms. Future progress will likely depend on identifying which aspects of consciousness are explained by each framework rather than seeking a single theory capable of accounting for all conscious phenomena.

Despite their differences, contemporary theories of consciousness share several commonalities. Most agree that consciousness involves integration across multiple brain regions and levels of processing. Whether this integration occurs primarily in the posterior cortex, as in IIT, or the prefrontal cortex, as in GNWT, is still debated. Similarly, the role of recurrent processing is emphasized in both RPT and DIT, albeit at different spatial scales. What emerges is the possibility that these theories are not mutually exclusive but address different facets of the same phenomenon. For example, GNWT may explain access consciousness and cognitive control, while IIT and RPT focus on the core experience of awareness. DIT provides a cellular substrate, and PP offers a computational framework (Table 3).

An integrative approach would involve specifying the conditions under which different mechanisms contribute to consciousness. This may require a multiscale model incorporating cellular, network, and system-level processes. Such a model would acknowledge that consciousness is not a monolithic entity but a dynamic, multifaceted process. Future research should aim at empirically testing the predictions of each theory in overlapping experimental paradigms. Cross-disciplinary collaborations, including philosophy, computational modeling, and clinical neuroscience, will be essential in refining our understanding.

## 6. Open Questions, Limitations, and Future Directions

Despite substantial progress in identifying neural correlates and computational mechanisms associated with conscious experience, several fundamental questions remain unresolved. Most contemporary neuroscientific theories successfully explain aspects of information processing, perception, attention, memory, and behavioral report. However, the relationship between these neural processes and the subjective qualities of experience—the so-called hard problem of consciousness—remains controversial. Whether phenomenal experience can ultimately be explained within existing neurobiological frameworks or requires new conceptual approaches continues to be a central topic of debate [86,87].

Notably, Hameroff and Penrose have emphasized that even unicellular organisms such as *Physarum polycephalum* (slime mold) can navigate mazes and solve problems, while *Paramecium* demonstrates intelligent behaviors including swimming, locating food and mates, learning, memory, and sexual reproduction—all in the absence of synaptic connections or a nervous system [88,89]. These phenomena suggest that intelligent, goal-directed behavior and rudimentary forms of information processing occur outside the conventional neural architecture. While these findings challenge strictly neuron-centric accounts, they are also widely interpreted as examples of sophisticated non-conscious information processing, suggesting that adaptive behavior does not necessarily require consciousness. Both interpretations remain under debate [85,90,91,92,93].

These unresolved issues highlight the importance of maintaining theoretical pluralism while preserving rigorous empirical standards. The continuing development of advanced neuroimaging, computational modeling, perturbational approaches, and theory-driven experimental paradigms may help discriminate among competing explanations. Rather than indicating a failure of existing neuroscience, current limitations may reflect the extraordinary complexity of consciousness itself and the need for integrative frameworks capable of linking subjective experience to mechanisms operating across multiple biological scales.

Together, these unresolved issues raise three particularly important questions: how conscious experience differs from sophisticated information processing and adaptive responsiveness; how phenomenal experience becomes available for cognition, memory, decision-making, and action; and whether consciousness requires a minimum degree or particular form of biological complexity.

### 6.1. Information Processing, Responsiveness, and Conscious Experience

A fundamental challenge in consciousness research is distinguishing information processing, adaptive responsiveness, and subjective experience. Complex systems can detect stimuli, integrate information, learn, optimize behavior, and produce context-sensitive responses without this necessarily demonstrating phenomenal consciousness. This distinction is relevant not only to artificial intelligence but also to non-neural biological systems and patients with disorders of consciousness, in whom behavioral responsiveness and conscious experience may dissociate. The absence of overt behavior does not necessarily indicate the absence of consciousness, as illustrated by covert consciousness in some clinically unresponsive patients; conversely, sophisticated behavioral output does not by itself establish the presence of subjective experience.

Different theories place the boundary between sophisticated information processing and consciousness at different points. GNWT emphasizes the global availability of information for flexible cognition and report, whereas RPT argues that recurrent sensory processing may be sufficient for phenomenal experience even without higher-order reportability. IIT is, in principle, substrate-independent and evaluates consciousness according to the intrinsic causal and informational structure of a system rather than its biological composition alone. Higher-order theories instead require a representation of one’s own mental states, whereas biological or physical theories such as DIT and Orch-OR assign indispensable roles to particular cellular or physical mechanisms. Consequently, the same artificial system could potentially satisfy consciousness-related criteria under one theoretical framework while failing to do so under another. Current AI systems therefore expose a fundamental unresolved question: whether functional sophistication and adaptive behavior are sufficient for consciousness, merely correlates of processes associated with it, or neither.

### 6.2. From Experience to Access, Decision-Making, and Action

Another unresolved mechanistic gap concerns how phenomenal experience becomes available for conscious access, decision-making, voluntary action, memory, and behavioral report. Existing theories frequently emphasize different stages of this process rather than providing a complete account of their interaction. RPT focuses primarily on the emergence of phenomenal content through recurrent sensory activity, GNWT on its global availability for cognition and report, MToC on its temporal organization and relationship with memory, and DIT on potential cellular mechanisms supporting integration. Whether these mechanisms represent successive components of a common architecture, partially independent processes, or mutually incompatible explanations remains unresolved. A comprehensive theory of consciousness will ultimately need to explain not only why experience occurs but also how experience is integrated with cognition, agency, memory, and behavior.

### 6.3. Is There a Minimum Level of Complexity Required for Consciousness?

A related unresolved question concerns whether a minimum level or particular form of biological complexity is necessary for consciousness to emerge. Current evidence does not establish a specific threshold based on neuron number, brain size, cortical organization, or computational complexity. Moreover, structural complexity alone may be insufficient, as different theories emphasize distinct organizational requirements, including recurrent connectivity, global information broadcasting, causal integration, higher-order representation, dendritic organization, or other substrate-specific mechanisms. Comparative neuroscience may therefore be particularly valuable in determining whether consciousness depends on a quantitative threshold of complexity or instead on particular qualitative features of biological organization. Until reliable independent markers of subjective experience are available, identifying the minimal architecture necessary for consciousness will remain an open empirical and philosophical question.

### 6.4. Strengths and Limitations of This Review

This review has several strengths. It adopts an explicitly integrative and multiscale perspective, comparing theories operating at cellular, network, computational, phenomenological, and physical levels while distinguishing differences in empirical support. It also incorporates recent adversarial testing of major consciousness theories and emphasizes the distinction between neural correlates, cognitive functions, adaptive behavior, and subjective experience.

Several limitations should nevertheless be acknowledged. First, this is a narrative rather than systematic review, and the selection of theories was intentionally illustrative rather than exhaustive. Consequently, several relevant frameworks receive limited discussion or fall outside the proposed organizational categories. Second, theories of consciousness frequently use inconsistent terminology and differ in their explananda, making direct comparison difficult. Third, the available empirical literature is heterogeneous and often relies on indirect markers of consciousness, task-dependent reports, or correlational neuroimaging findings. Finally, philosophical, neurobiological, computational, and physical theories cannot always be evaluated according to identical evidential standards. These limitations reinforce the need for clearly specified predictions, preregistered experiments, adversarial collaborations, and greater conceptual precision in future consciousness research.

### 6.5. Future Directions

Future research should prioritize experiments capable of generating genuinely divergent predictions between competing theories. Particularly important directions include preregistered adversarial collaborations; multimodal integration of electrophysiology, neuroimaging, perturbational techniques, and computational modeling; improved methods for detecting covert consciousness in clinically unresponsive patients; clearer operationalization of phenomenal versus access consciousness; and explicit criteria for evaluating consciousness claims in artificial and non-neural systems. Multiscale approaches linking dendritic physiology, recurrent circuit dynamics, large-scale broadcasting, information integration, memory, and behavior may also help determine whether current theories describe competing mechanisms or complementary components of a broader architecture.

## 7. Conclusions

No contemporary theory has yet provided a complete explanation of consciousness. Leading frameworks differ not only in their proposed neural mechanisms but also in what they attempt to explain: phenomenal experience, cognitive access, reportability, information integration, memory, or cellular and physical mechanisms. Several fundamental questions therefore remain unresolved. What distinguishes conscious experience from sophisticated but unconscious information processing? Is consciousness necessarily dependent on biological neural tissue, or could sufficiently organized artificial systems possess it? How does subjective experience become available for memory, decision-making, voluntary action, and reporting? What degree or form of biological complexity is necessary for consciousness to emerge?

Progress will require clearer terminology, preregistered theory-driven experiments, direct adversarial comparisons, improved assessment of covert consciousness, and multiscale models capable of linking cellular, network, computational, and phenomenological levels of explanation. Rather than assuming that one existing theory will necessarily displace all others, future research should determine which components of consciousness each framework can genuinely explain and where critical explanatory gaps remain. The central challenge is therefore no longer merely to identify neural activity associated with consciousness but to establish why and under what conditions particular physical processes are accompanied by subjective experience.

## Figures and Tables

**Table 1:** Key terms used in consciousness research.

Term	Operational Definition Used in This Review	Important Distinction
Consciousness	Broad presence of subjective experience	Umbrella concept; exact boundaries remain disputed
Phenomenal consciousness	Qualitative first-person character of experience; what an experience feels like	Does not necessarily require reportability or explicit self-reflection
Access consciousness	Information available for reasoning, report, decision-making, and voluntary behavior	May involve global availability without resolving why subjective experience occurs
Awareness	Context-dependent detection or experience of internal or external stimuli	Used inconsistently in the literature; not always equivalent to consciousness
Subjective experience	First-person experiential character of a mental state	Closely overlaps with phenomenal consciousness
Self-consciousness	Awareness or representation of oneself as the subject of experience	Not necessarily intrinsic to every conscious experience
Neural correlates of consciousness	Minimal neural mechanisms jointly sufficient for a particular conscious experience or conscious state	Correlation alone does not establish a complete causal explanation of consciousness

**Table 2:** Organizational roadmap and relative empirical status of the principal frameworks discussed in this review.

Framework	Primary Explanatory Level	Principal Focus	Empirical Status	Main Manuscript Section
GNWT	Large-scale network	Access and global broadcasting	Strong empirical engagement	3.1
HOT	Higher-order cognitive	Meta-representation	Moderate/debated	3.1
RPT	Local recurrent network	Phenomenal consciousness	Strong empirical engagement	3.1
MToC	Memory systems	Temporal reconstruction and memory	Moderate/emerging	3.1
IIT	Information-theoretical/system	Integrated experience	Moderate and debated	3.1
DIT	Cellular	Dendritic integration	Emerging	3.1
PP	Computational	Prediction and generative models	Strong framework; consciousness-specific claims debated	3.1
Field-based theories	Physical field	Integration/unification through endogenous fields	Speculative/emerging	3.4
Orch-OR	Quantum/physical	Quantum state reduction	Highly speculative	3.3
Dualism/ panpsychism	Philosophical/ontological	Nature of mind and experience	Philosophical/metaphysical	3.2

Abbreviations: DIT, Dendritic Integration Theory; GNWT, Global Neuronal Workspace Theory; HOT, Higher-Order Theory; IIT, Integrated Information Theory; MToC, Memory Theory of Consciousness; PP, Predictive Processing; RPT, Recurrent Processing Theory.

**Table 3:** Relative empirical and methodological status of theories discussed in this review.

Theory	Primary Focus	Neural Substrate	Consciousness Type	Empirical Markers	Empirical Evidence/Challenges
**GNWT** (Global Neuronal Workspace Theory)	Access	Frontoparietal cortex	Access consciousness	P3b, fMRI ignition	Partial support; limited evidence for sustained “ignition” at stimulus offset; prefrontal involvement debated [85]
**IIT** (Integrated Information Theory)	Phenomenal	Posterior hot zone	Phenomenal consciousness	PCI, Φ (Phi) estimates	Challenged posterior connectivity predictions; lack of consistent evidence for sustained integration patterns [85]
**RPT** (Recurrent Processing Theory)	Phenomenal	Visual cortex	Phenomenal consciousness	VAN, local recurrence	Not directly tested in COGITATE; supported by evidence for recurrent processing in perception
**DIT** (Dendritic Integration Theory)	Micro-scale integration	Layer 5 pyramidal neurons	Mixed (phenomenal & access)	Dendritic spikes (theoretical)	Largely theoretical; limited direct empirical validation
**PP** (Predictive Processing)	Computational modeling	Hierarchical neural networks	Both phenomenal & access	Prediction errors, precision-weighting	Not directly tested in COGITATE; supported by computational and behavioral evidence
**HOT** (Higher-Order Theories)	Meta-awareness	Prefrontal cortex	Access consciousness	Metacognition, confidence ratings	Empirical support debated; challenges regarding the necessity of higher-order representations
**MToC** (Memory Theory of Consciousness)	Reconstructed memory	Hippocampus, related cortex	Phenomenal consciousness	Postdictive effects, amnesia patterns	Critiqued for potential conflation of memory and consciousness

## Data Availability

No new data were created or analyzed in this study. Data sharing is not applicable to this article.
